# Levels and patterns of objectively-measured physical activity volume and intensity distribution in UK adolescents: the ROOTS study

**DOI:** 10.1186/1479-5868-11-23

**Published:** 2014-02-24

**Authors:** Paul J Collings, Katrien Wijndaele, Kirsten Corder, Kate Westgate, Charlotte L Ridgway, Valerie Dunn, Ian Goodyer, Ulf Ekelund, Soren Brage

**Affiliations:** 1 MRC Epidemiology Unit, University of Cambridge, Cambridge, UK; 2 Developmental Lifecourse Research Group, Department of Psychiatry, University of Cambridge, Cambridge, UK; 3 Department of Sport Medicine, Norwegian School of Sports Science, Oslo, Norway; 4 Physical Activity Programme, MRC Epidemiology Unit, Addenbrookes Hospital, University of Cambridge, Institute of Metabolic Science, Box 285, Cambridge CB2 0QQ, UK

**Keywords:** Energy expenditure, Physical activity intensity, Sedentary time, Activity monitoring, Adolescents

## Abstract

**Background:**

Few studies have quantified levels of habitual physical activity across the entire intensity range. We aimed to describe variability in total and intensity-specific physical activity levels in UK adolescents across gender, socio-demographic, temporal and body composition strata.

**Methods:**

Physical activity energy expenditure and minutes per day (min/d) spent sedentary and in light, moderate, and vigorous intensity physical activity were assessed in 825 adolescents from the ROOTS study (43.5% boys; mean age 15.0 ± 0.30 years), by 4 days of individually calibrated combined heart rate and movement sensing. Measurement days were classified as weekday or weekend and according to the three school terms: summer (April-July), autumn (September-December), and spring (January-March). Gender and age were self-reported and area-level SES determined by postcode data. Body composition was measured by anthropometry and bio-electrical impedance. Variability in physical activity and sedentary time was analysed by linear multilevel modelling, and logistic multilevel regression was used to determine factors associated with physical inactivity (<60 min moderate-to-vigorous intensity physical activity/d).

**Results:**

During awake hours (15.8 ± 0.9 hrs/d), adolescents primarily engaged in light intensity physical activity (517 min/d) and sedentary time (364 min/d). Boys were consistently more physically active and less sedentary than girls, but gender differences were smaller at weekends, as activity levels in boys dropped more markedly when transitioning from weekday to weekend. Boys were more sedentary on both weekend days compared to during the week, whereas girls were more sedentary on Sunday but less sedentary on Saturday. In both genders light intensity physical activity was lower in spring, while moderate physical activity was lower in autumn and spring terms, compared to the summer term; sedentary time was also higher in spring than summer term. Adolescents with higher fatness engaged in less vigorous intensity physical activity. Factors associated with increased odds of physical inactivity were female gender, both weekend days in boys, and specifically Sunday in girls.

**Conclusions:**

Physical activity components vary by gender, temporal factors and body composition in UK adolescents. The available data indicate that in adolescence, girls should be the primary targets of interventions designed to increase physical activity levels.

## Introduction

Moderate-to-vigorous intensity physical activity (MVPA) has consistently been associated with numerous physiological and psychological health benefits in children and adolescents [1-3]. It is therefore recommended by UK government [4] and other agencies [5,6] that youth participate in a minimum 60 minutes of MVPA per day (min MVPA/d) for maintenance of general health. Traditionally, the foremost objective of studies reporting on levels and patterns of physical activity performed by adolescents has been to determine the amount of coherence between recommended and actual MVPA levels [7-11]. Many studies have also investigated the correlates of MVPA, which include gender, parental education, and a host of psychological, social and cultural factors [12]. Knowledge of this type is important as it can be used to increase MVPA participation by aiding the design of effective interventions tailored to specific groups. However, recent evidence has indicated that physical activity performed at other intensities (e.g., light and vigorous) may exhibit unique associations with health, and thus measurement and investigation of MVPA alone may now be considered inadequate [13].

The health-related outcomes of light intensity physical activity are of increasing research interest [14], and at the same time the concept that vigorous intensity physical activity may confer additional health benefits beyond physical activity of moderate intensity is gathering support [15]. As a direct consequence of the latter, guidelines now explicitly recommend vigorous intensity physical activity on at least three days per week, but no overall activity volume is specified for the vigorous component, perhaps partly due to insufficient data [4-6]. Whilst there has been increasing research and policy emphasis on the health benefits of activity intensities other than MVPA, few studies have utilised objective monitoring tools in adolescents to describe the levels and correlates of habitual physical activity participation throughout the entire range of activity intensities [16-19].

Occupying the lowest end of the intensity spectrum, sedentary behaviour is defined as any waking behaviour in a sitting or reclining posture with an energy expenditure <1.5 times the resting metabolic rate [20]. It is thought to be associated with components of the metabolic syndrome (particularly higher body fatness), lower fitness, and lower self-esteem in school-aged children and youth [21]. Like the evidence-base for vigorous activity, however, the relatively premature nature of this research area has not yet permitted specific recommendations to be made regarding overall sedentary time, other than in the broadest sense of limiting daily sedentary behaviour [4]. Whilst increasing numbers of studies have described total sedentary time in adolescents [7-9,16,18,19,22-24], more are needed to examine the correlates and determinants of this highly prevalent behaviour [12].

This study was conducted to describe gender-specific levels of total habitual physical activity energy expenditure (PAEE), the time spent in different physical activity intensities (light, moderate and vigorous) and sedentary time in a sample of healthy UK adolescents. We further investigated socio-demographic and physical correlates of physical activity and sedentary behaviour, as well as the temporal patterns of these behaviours.

## Methods

### Physical activity assessment

The ROOTS study is a longitudinal investigation of risk factors for adolescent psychopathology that is described in detail elsewhere [25]. At wave 0, 1238 adolescents were recruited from 18 schools in the East of England, of which 1203 students (aged 14.5 ± 0.28 years) attended for testing. About six months after wave 0 measurements, 930 participants (75% of the original cohort; 15.0 ± 0.31 years) accepted an invitation to undergo monitoring of habitual physical activity at wave 1. All procedures were explained prior to their conduct and participants could choose to decline any part of the study. The ROOTS study was approved by the Cambridge research ethics committee.

Wave 1 entailed fitting participants with a combined heart rate and movement sensor (Actiheart, CamNtech Ltd, Papworth, UK), a small waterproof device that can be worn continuously during free-living to provide estimates of activity intensity in youth [26,27]. This was attached to the participant’s chest by two ECG electrodes, one placed medially at the base of the sternum and the other horizontally to the left side without the adjoining wire being too taught [28].

A graded 8-min sub-maximal step-test (150 mm high step) was conducted to individually calibrate heart rate to protocol-estimated physiological intensity for the free-living activity assessment [29]. Upon conclusion of the step-test the combined sensor was initialised to record data in 30 second epochs and participants were requested to wear the monitor continuously for 4 consecutive days. To incorporate week and weekend days, monitors were typically fitted on a Friday and retrieved early the following week. Subsequently, heart rate data were cleaned [30] and individually calibrated with parameters from the step test, and combined with trunk acceleration [29] to derive an estimated activity intensity (J/min/kg) time-series via branched equation modelling [31]. Step-test data were considered valid if at least 4-min of the protocol were completed. For participants without a valid step test but with valid free-living data (*n* = 65), a group calibration equation was derived on the basis of all valid step tests in the sample, representing the average calibration curve for a given age, gender and sleeping heart rate level. Average daily PAEE (kJ/kg/day) was subsequently derived by integration of the intensity time-series with respect to time (area under the curve). Non-wear segments were inferred from the combination of prolonged periods of zero acceleration accompanied by non-physiological heart rate data, and data were adjusted to minimise potential diurnal bias during summarisation. The time distribution of activity intensity was described by summarising the intensity time-series in standard metabolic equivalents (METs), within 18 narrowly defined intensity categories. For tabulation purposes these categories were later collapsed into broader intensity categories as sedentary (≤1.5 METs), light intensity physical activity (1.5 to 4 METs), moderate intensity physical activity (4 to 7 METs), and vigorous intensity physical activity (>7 METs). These broader MET thresholds have been commonly applied when investigating physical activity in children and youth [15].

**Figure 1:**
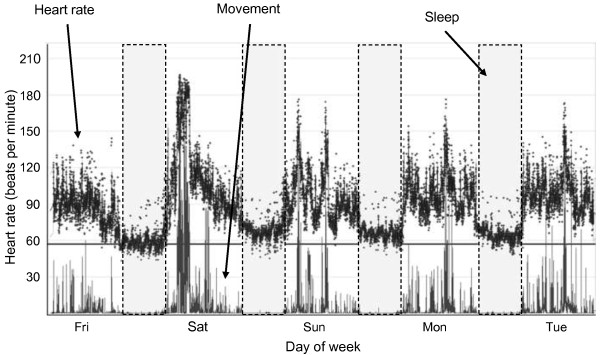
**Separation of awake sedentary time and sleep.** Self-reported bed times (grey blocks) were overlaid upon movement and heart rate data and manipulated (if necessary) to coincide with objective markers of sleep. Markers of sleep initiation were the beginning of sustained low levels of movement and a steady decline in heart rate, markers of awakening were movement initiation after long periods of very little movement, together with an abrupt increase in heart rate. In this example, the sensor was attached on Friday and removed the following Tuesday.

Protocols that involve continuous wear periods are advantageous as they limit missing data. However, they are susceptible to misclassification of awake sedentary time and sleep, which are difficult to distinguish from one another solely on the basis of heart rate and trunk acceleration data. In this study, adolescents were asked to report the times that they usually went to bed and got up on week and weekend days. This information was overlaid on the combined heart rate and movement time-series plot to provide an initial classification of data into asleep/awake blocks. All time-series plots were visually inspected and when necessary these blocks were adjusted to coincide with features within the objective data (Figure 1). This approach of fusing objective and subjective data has been shown to improve classification accuracy of sleep detection [32].

Individuals were included in the present study if they contributed at least one day of valid activity data, and person-days were used for the main analysis to enable comparisons of physical activity patterns across week and weekends day. A valid day was defined as ≥16 hours of observation time, with these hours being roughly equally distributed between the morning (3 am-9 am), noon (9 am-3 pm), afternoon (3 pm-9 pm), and midnight (9 pm-3 am) parts of the day (i.e. ≥4 hours in each of these quadrants of the day). These criteria resemble the precedent that 70% of a whole day when implementing continuous wear protocols is necessary for valid person-day level activity data [22,33].

### Other data collection

The real-time stamps of the combined sensor records were used to categorise days as either weekday (Monday-Friday) or weekend (Saturday and Sunday) and according to the three school terms: summer (April-July), autumn (September-December), and spring (January-March).

A demographic questionnaire was used to collect information regarding participant gender, date of birth, ethnicity (White, other) and postcode. The latter was used to generate an area-level SES variable according to the ‘A classification of residential neighbourhoods’ (ACORN) index [34]. This index classifies UK addresses into one of five main SES groups: hard-pressed, moderate means, comfortably off, urban prosperity, or wealthy achievers. The two lowest categories (hard-pressed and moderate-means) were combined as were the two highest categories (urban prosperity and wealthy achievers) to create a trichotomous low, middle and high area-level SES variable.

Menarcheal status was self-reported upon enrolment to the ROOTS study and post-menarcheal girls were defined as pubertal. Girls yet to experience menarche were classified according to self-reported breast and pubic hair development using the Tanner image assessment method [35]; girls with breast or pubic hair stages ≥3 were categorised as pubertal. Likewise, boys self-reporting stages ≥4 on pubic hair coverage and genital development were defined as pubertal, whereas boys with stages ≤2 on both measures were classified as pre-pubertal. Remaining boys were categorised according to salivary testosterone concentrations (>25^th^ percentile level from the pubertal group corresponded to pubertal).

Height (cm) and weight (kg) were measured by standard anthropometric procedures and the body mass index was calculated (BMI, kg/m^2^). Body composition and body tissue impedance were also estimated using the Tanita TBF-300 MA bioelectrical impedance analyser. Estimates of body fatness were subsequently calculated by using a number of child-specific prediction algorithms based on height and weight [36-38], BMI [39-41], or impedance data [42]. All permutations were pooled to yield an aggregated measure of fat mass (kg), which was subsequently converted to fat mass index (kg/m^2^) by division of stature squared [43,44]. Age- and gender-specific fat mass index cutoffs were generated with ≥85^th^ percentile used to denote overfat adolescents (<15.0 years: boys ≥4.49 kg/m^2^, girls ≥7.48 kg/m^2^; >15.0 years: boys ≥4.86 kg/m^2^, girls ≥7.98 kg/m^2^). Fat mass index was preferred to percentage body fat for derivation of body fat status as per the most recent UK paediatric reference data [45].

### Statistical analysis

Sample characteristics were computed for the whole sample and stratified by gender. Gender differences were assessed using independent sample t-tests, Wilcoxon-Mann–Whitney tests, and Chi-square tests for normally distributed, skewed, and categorical data, respectively. The same tests were used to compare participants included versus those excluded from the study due to missing data.

Gender differences in continuous MET-intensity distributions were examined using a multivariate test of means. To investigate crude levels of total physical activity, the time spent in the broader activity intensity categories and sedentary time (in addition to correlations between variables), daily data were collapsed to the average-daily level and unadjusted mean summary estimates were calculated by standard linear regression, accounting for school-level clustering. Daily-level data were retained for the main analyses.

To explore associations between socio-demographic, temporal and body composition factors with physical activity and sedentary time, linear multilevel modelling techniques were used (one model for each dependent variable). All potential correlates (gender, type of day, school term, area-level SES, body fat status) were included simultaneously in models to achieve mutual adjustment. Age was also included in models as a control variable, but ethnicity and pubertal status were not included due to low variation within the sample (>90% White; >90% pubertal at wave 0) and some missing data. Interaction terms between gender and all other potential correlates (gender*correlate) were added to models but were only retained if Wald tests proved significant (in addition to likelihood ratio tests for variables with >2 levels). All results are presented as adjusted means and confidence intervals. Prior to multilevel analyses moderate and vigorous intensity physical activity were both natural log-transformed to approximate normal distributions. Their data have been exponentiated back to the original scale for interpretation purposes. To determine factors related to the odds of being inactive (<60 min MVPA/d), a logistic multilevel model was constructed using an analogous approach to linear models. In all multilevel models (linear and logistic), intercepts were allowed to vary randomly between participants to account for clustering of data at the participant level by virtue of repeated days of physical activity measurement. A second level to account for school-level clustering was also included. A significance level of *p* < 0.05 was chosen a priori and all analyses were performed with Stata 13.0 software (StataCorp, College Station, TX).

## Results

One-hundred and five participants (11.3%) did not provide valid activity data, leaving a final sample size of 825 participants with mean age 15 (± 0.30) years for the present analyses, as described in Table 1. The sample contained fewer boys (43.5%) than girls, and was predominantly composed of participants from higher SES areas. Boys were taller, heavier and leaner than girls. Boys also slept less than girls, and supplementary analyses showed that for both genders sleep durations were longer on Saturday and Sunday compared to weekdays (*p* < 0.001). The 105 participants excluded from the study due to missing data had higher BMI (21.0 vs. 20.1 kg/m^2^, *p* = 0.024), fat mass (13.3 vs. 11.5 kg, *p* = 0.045) and fat mass index (4.7 vs. 4.2 kg/m^2^, *p* = 0.033), but did not otherwise differ from those who contributed to the analysis (*p* ≥ 0.08).

**Table 1:** Descriptive characteristics of the study population

	**Whole sample (*n*** **= 825)**	**Boys (*n*** **= 359)**	**Girls (*n*** **= 466)**	***p*-gender difference**
Age (y)	15.0 ± 0.30^*1*^	15.0 ± 0.31	15.0 ± 0.29	0.87
Pubertal status (*n* (%))^*2,3*^				
Pre-pubertal	79 (9.8)	71 (20.7)	8 (1.7)	
Pubertal	730 (90.2)	272 (79.3)	458 (98.3)	<0.001
Ethnicity (*n* (%))^*4*^				
White	767 (94.6)	341 (95.8)	426 (93.6)	
Other	44 (5.4)	15 (4.2)	29 (6.4)	0.18
Area-level SES (*n* (%))				
Low	124 (15.0)	62 (17.3)	62 (13.3)	
Middle	193 (23.4)	80 (22.3)	113 (24.3)	
High	508 (61.6)	217 (60.5)	291 (62.5)	0.27
Height (cm)	166.5 ± 7.9	171.4 ± 7.7	162.8 ± 5.8	<0.001
Weight (kg)	55.7 (13.3)^*5*^	57.8 (13.6)	53.9 (12.3)	<0.001
BMI (kg/m^2^)	20.1 (3.9)	19.7 (3.5)	20.3 (4.1)	0.0011
Fat mass (kg)	11.5 (7.8)	8.2 (5.3)	13.7 (6.9)	<0.001
Fat mass index (kg/m^2^)	4.2 (3.0)	2.8 (1.8)	5.2 (2.5)	<0.001
Overfat (%)^*6*^	123 (14.9)	53 (14.8)	70 (15.0)	0.92
Sleep duration (min/d)	493.7 ± 52.8	487.9 ± 48.8	498.2 ± 55.4	<0.01

^*1*^Mean ± SD (all such values) and gender comparisons performed by independent sample t-tests; ^*2*^Due to missing data based on 809 participants (343 boys and 466 girls); ^*3*^For categorical variables gender comparisons were made by chi-square tests; ^*4*^Due to missing data based on 811 participants (356 boys and 455 girls); ^*5*^Median and IQR in parentheses (all such values) and gender comparisons performed by Wilcoxon-Mann–Whitney tests; ^*6*^ ≥85^th^ percentile of age- and gender-specific fat mass index.

Participants contributed a total of 2381 valid person-days of physical activity data (mean 2.9, range 1–5 days), composed of 1256 weekdays (Wednesdays and Thursdays were underrepresented, <100 person-days each), 559 Saturdays and 566 Sundays. Participants were generally compliant with the continuous monitoring protocol; on average valid days included 23.8 hours of data with ≥5.8 hours disseminated within each of the quadrants of the day (morning, noon, afternoon, and night). Each of the school terms were represented with 648 (27.2%) spring, 1157 (48.6%) summer, and 576 (24.2%) autumn person-days, respectively.

**Figure 2:**
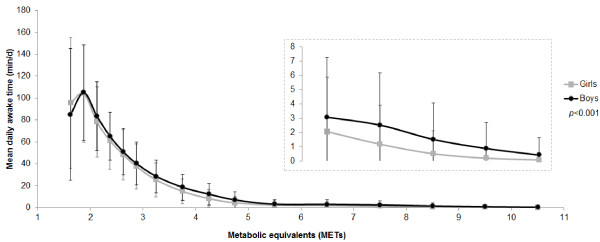
**Daily time (means and standard deviations) spent in 16 MET categories stratified by gender.** Light intensity physical activity corresponds to the region: 1.5 to 4 METs; Moderate intensity physical activity: 4 to 7 METs; Vigorous intensity physical activity: >7 METs. Inset shows magnified plot for 6 to 11 METs so that gender differences are observable. For scaling purposes data for time spent sedentary (≤1.5 METs) have been omitted and are reported both in-text and in Table 2.

Figure 2 shows the mean awake time spent in 16 MET categories stratified by gender (data points for ≤1.25 METs and 1.25 to 1.5 METs have been omitted for illustrative purposes). Boys and girls predominantly engaged in light intensity physical activity (1.5 to 4 METs), with considerably less moderate (4 to 7 METs) and vigorous (>7 METs) intensity physical activity. Apart from the very light intensity region (1.5 to 2 METs) boys were consistently more active than girls across the physical activity intensity range (*p* < 0.001).

Table 2 summarises the average daily levels of PAEE, and the minutes per day spent in the broader physical activity intensity categories and sedentary time. It shows that participants predominantly engaged in light intensity physical activity, and that levels of sedentary time were also substantial (364 min/d). The table further elaborates on Figure 2 by revealing that boys exhibited higher PAEE and lower sedentary time than girls. In terms of meeting or not meeting MVPA recommendations, a significantly lower proportion of boys were inactive (performing on average <60 min MVPA/d) compared to girls.

**Table 2:** Unadjusted average-daily physical activity and sedentary time estimates (means and 95% confidence intervals)

	**Whole sample (*n*** **= 825)**	**Boys (*n*** **= 359)**	**Girls (*n*** **= 466)**	***p*-gender difference**
Physical activity energy expenditure (kJ/kg/d)^*1*^	73.6 (70.0 to 77.1)	83.5 (80.2 to 86.9)	65.9 (61.9 to 69.9)	<0.001
Light physical activity (min/d)^*1*^	516.5 (495.1 to 537.9)	527.9 (506.3 to 549.6)	507.7 (482.7 to 532.8)	0.0037
Moderate physical activity (min/d)^*1*^	37.7 (33.4 to 42.4)	50.0 (43.7 to 57.2)	30.3 (26.2 to 35.0)	<0.001
Vigorous physical activity (min/d)^*1*^	3.4 (2.5 to 4.6)	8.4 (6.6 to 10.8)	1.7 (1.2 to 2.4)	<0.001
Sedentary time (min/d)^*1*^	363.9 (348.4 to 379.6)	338.4 (322.9 to 353.8)	383.7 (363.9 to 403.5)	<0.001
<60 min MVPA/d (*n* (%))^*2*^	448 (54.3)	131 (36.5)	317 (68.0)	<0.001

Data within person was collapsed to average values across days before analysis. Moderate and vigorous physical activity were transformed using natural log as these variables were skewed before analysis; presented values have been exponentiated back to the original scale. ^*1*^Gender comparisons made by linear regression accounting for within-school clustering; ^*2*^Gender comparison made by chi-square test.

Table 3 contains bivariate correlation coefficients between each of the physical activity variables and sedentary time. All physical activity variables were positively correlated, and time spent sedentary was inversely correlated, with PAEE. The magnitude of all coefficients were largely equivalent between genders, except for light intensity physical activity which was more strongly correlated with PAEE (*r* = 0.65 vs. 0.42) and moderate intensity physical activity (*r* = 0.30 vs. 0.15) in girls. In contrast, vigorous intensity physical activity was more strongly correlated with PAEE in boys than girls (*r* = 0.75 vs. 0.63).

**Table 3:** Pearson correlations between average-daily PAEE and the physical activity subcomponents

		**PAEE**	**Light physical activity**	**Moderate physical activity**	**Vigorous physical activity**
Boys	Light physical activity	0.42	-	-	-
	Moderate physical activity	0.84	0.15	-	-
	Vigorous physical activity	0.75	0.07^*1*^	0.52	-
	Sedentary time	−0.73	−0.83	−0.51	−0.29
Girls	Light physical activity	0.65	-	-	-
	Moderate physical activity	0.84	0.30	-	-
	Vigorous physical activity	0.63	0.04^*2*^	0.52	-
	Sedentary time	−0.78	−0.88	−0.52	−0.27

Data within person was collapsed to average values across days before analysis. PAEE, Physical activity energy expenditure. For all correlations *p* < 0.001 unless otherwise specified. ^*1*^*p* = 0.26; ^2^*p* = 0.49.

There was a significant interaction between gender and type of day (*p* < 0.001 for multilevel models including sedentary time, light intensity physical activity, vigorous intensity physical activity, and PAEE), but all other interactions with gender were not statistically significant (*p* > 0.05). Therefore, multilevel linear models for type of day were analysed stratified by gender, whilst the results for all the other variables are taken from models inclusive of a gender by type-of-day interaction term (Table 4). In agreement with unadjusted estimates the multilevel models showed that boys were generally more physically active and less sedentary than girls. However, compared to weekdays, boys engaged in less PAEE and were less active across all intensities of physical activity at the weekend; additional pairwise comparisons revealed that boys were less moderately active on Sunday versus Saturday. Sedentary time in boys was also higher on both weekend days by >60 min/d. In girls, while there were no differences in PAEE and vigorous activity between weekdays and Saturday, Saturdays were characterised by lower levels of light and moderate physical activity. This difference in light activity was less marked than in boys however, and manifested as girls performing more light intensity physical activity than boys on Saturday. Similarly to boys, girls had lower levels of PAEE and were less active across all intensities of physical activity on Sunday compared to weekdays. However, contrasting boys, girls were less physically active in terms of total volume and across all intensities of activity on Sunday relative to Saturday. Furthermore, girls’ awake sedentary time was lower on Saturday and higher on Sunday compared to during the week, by approximately 20 min/d, and awake sedentary time was also higher on Sunday than Saturday. With regards school term, light and moderate intensity physical activities were lower, and sedentary time higher by approximately 30 min/d in spring term compared to summer term. Moderate intensity physical activity was also lower in autumn than summer term. Children residing in middle SES areas performed approximately 30 min/d more light intensity physical activity and 30 min/d less sedentary time than children from low SES areas. Whilst, compared to children within the normal body fat range, overfat adolescents (≥85^th^ percentile) engaged in less vigorous physical activity per day.

**Table 4:** Correlates of daily physical activity and sedentary time (adjusted means and 95% confidence intervals)

	**PAEE (kJ/kg/d)**^***2***^	***p*-value**	**Light PA (min/d)**	***p*-value**	**Moderate PA (min/d)**	***p*-value**	**Vigorous PA (min/d)**	***p*-value**	**Sedentary time (min/d)**	***p*-value**
Boys										
Ref: Weekday	90.5 (87.1 to 93.9)		575.3 (559.8 to 590.8)		52.7 (46.7 to 59.6)		5.0 (3.9 to 6.3)		308.2 (294.1 to 322.2)	
Saturday	77.8 (73.3 to 82.4)	<0.001	484.2 (466.1 to 502.4)	<0.001	31.6 (26.7 to 37.3)	<0.001	2.7 (1.9 to 3.6)	<0.001	372.4 (354.3 to 390.5)	<0.001
Sunday	74.2 (69.7 to 78.6)	<0.001	468.2 (450.2 to 486.2)	<0.001	21.4 (18.1 to 25.2)^*1*^	<0.001	2.4 (1.7 to 3.2)	<0.001	375.0 (357.2 to 392.8)	<0.001
Girls										
Ref: Weekday	69.1 (65.9 to 72.2)		533.0 (517.0 to 549.0)		28.0 (24.1 to 32.5)		0.9 (0.7 to 1.1)		384.5 (369.6 to 399.4)	
Saturday	70.3 (66.4 to 74.2)	0.517	510.3 (491.6 to 529.0)	0.005	16.0 (13.1 to 19.4)	<0.001	0.8 (0.6 to 1.0)	0.329	362.2 (343.7 to 380.7)	0.014
Sunday	55.4 (51.6 to 59.3)^*1*^	<0.001	444.4 (425.7 to 463.2)^*1*^	<0.001	9.2 (7.5 to 11.2)^*1*^	<0.001	0.5 (0.4 to 0.7)^*2*^	<0.001	403.5 (385.0 to 422.0)^*1*^	0.036
School term										
Ref: Summer	76.0 (72.4 to 79.6)		526.2 (508.6 to 543.8)		29.2 (25.5 to 33.5)		1.5 (1.2 to 1.9)		353.8 (339.2 to 368.3)	
Autumn	72.7 (67.3 to 78.0)	0.310	520.5 (494.0 to 547.0)	0.726	21.3 (17.4 to 26.2)	0.012	1.7 (1.2 to 2.4)	0.619	363.2 (341.5 to 384.9)	0.481
Spring	70.7 (66.0 to 75.3)	0.068	490.5 (467.9 to 513.2)	0.012	23.1 (19.3 to 27.7)	0.042	1.3 (1.0 to 1.7)	0.326	385.0 (365.8 to 404.2)	0.010
Area-level SES										
Ref: Low	73.0 (68.2 to 77.7)		499.7 (477.0 to 522.3)		23.1 (18.8 to 28.4)		1.4 (1.0 to 2.0)		377.5 (355.3 to 399.7)	
Middle	73.9 (69.9 to 77.9)	0.740	530.1 (511.1 to 549.2)	0.022	27.2 (23.0 to 32.3)	0.208	1.5 (1.1 to 1.9)	0.873	349.0 (330.7 to 367.3)	0.042
High	73.9 (71.0 to 76.8)	0.716	513.6 (499.5 to 527.6)	0.238	25.4 (22.6 to 28.5)	0.412	1.5 (1.3 to 1.9)	0.623	367.0 (354.7 to 379.2)	0.393
Body fat status^*3*^										
Ref: Normal	73.9 (71.3 to 76.4)		515.3 (502.7 to 527.9)		25.5 (23.1 to 28.1)		1.6 (1.3 to 1.8)		364.0 (353.7 to 374.4)	
Overfat	72.2 (66.5 to 77.8)	0.539	512.6 (488.3 to 536.9)	0.816	24.4 (18.9 to 31.5)	0.734	1.0 (0.7 to 1.5)	0.022	370.8 (345.4 to 396.3)	0.596

Statistical comparisons were made by multilevel modelling accounting for repeated-measures and within-school clustering. All factors mutually adjusted for one another and age. PAEE, Physical activity energy expenditure. Moderate and vigorous physical activity were transformed using natural log as these variables were skewed before analysis; presented values have been exponentiated back to the original scale. ^*1*^Significantly different to Saturday, *p* < 0.001; ^*2*^Significantly different to Saturday, *p* = 0.015; ^*3*^Normal: <85^th^ percentile of age- and gender-specific fat mass index, Overfat: ≥85^th^ percentile of age- and gender-specific fat mass index.

**Figure 3:**
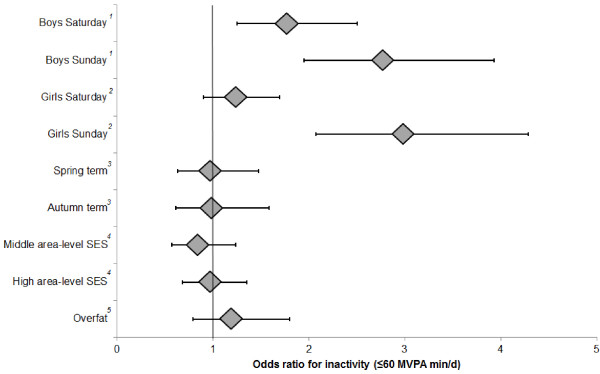
**Adjusted odds ratios (and 95% confidence intervals) for physical inactivity (<60 min MVPA/d).** All factors mutually adjusted for one another and controlled for age. Reference categories: ^*1*^Boys weekday activity; ^*2*^Girls weekday activity; ^*3*^Summer term; ^*4*^Low area-level SES; ^*5*^Normal fat (<85^th^ percentile of age- and gender-specific fat mass index).

Figure 3 provides information regarding odds ratios for physical inactivity (accumulating <60 min MVPA/d). To be consistent with linear models, the data pertaining to type of day are from gender-stratified logistic multilevel models, whereas the results for all other variables are from whole-sample models incorporating a gender by type-of-day interaction term. Boys were more likely to be inactive on Saturday (OR = 1.77, 95% CI 1.25-2.50, *p* = 0.001) and Sunday (2.77, 1.95-3.93, *p* < 0.001) compared to weekdays. Girls on the other hand were more likely to be inactive on Sunday only (2.98, 2.08-4.29, *p* < 0.001)). Additional pairwise comparisons showed that both genders were more likely to be inactive on Sunday compared to Saturday (*p* ≤ 0.026). Aligning with the data in Tables 2 and 4, further comparisons also showed that girls had significantly higher odds of physical inactivity on all types of day compared to boys (*p* < 0.001). For instance, girls were 3.6 times more likely to be inactive on weekdays than boys (95% CI 2.44- 5.94).

## Discussion

### Levels of physical activity and sedentary time

On average PAEE for the whole sample was 74 kJ/kg/d, which is comparable to levels reported in other countries [46-48]. Unadjusted estimates also showed that boys expended nearly 18 kJ/kg/d more energy in activity than girls; an observation that corroborates work in Dutch adolescents [48]. In terms of intensity distributions, adolescents’ waking hours were dominated by light intensity physical activity which accounted for 51% of the waking day and contributed to 51% of total daily PAEE (38% contribution from MVPA and 11% from sedentary time). Gender differences in light intensity physical activity were modest at approximately 20 min/d. As the health benefits acquired from light intensity physical activity are at present largely unknown, the high prevalence of this exposure in both adolescent boys and girls implies that additional research surrounding this intensity is needed. Compared to the light intensity domain, levels of moderate and vigorous intensity physical activity were low at 38 and 3.4 min/d, respectively. These data are remarkably similar to levels reported in a sample of nationally representative 12–15 year old children taking part in the Health Survey for England [17]. As in this study, Health Survey for England also found that boys, compared to girls, participated in approximately 20 min/d more objectively-measured moderate intensity physical activity and several min/d more vigorous intensity physical activity. These data likely explain why light intensity physical activity was correlated with PAEE more strongly in girls than boys, as a greater proportion of PAEE was derived from light intensity physical activity in girls (55% vs. 47%). The data further indicate (assuming that vigorous intensity physical activity confers some unique health benefits [15]) that in adolescence there is a need to promote participation within the vigorous domain, particularly in girls.

Time spent sedentary in the current study was considerable at 364 min/d, but the Health Survey for England reported 509 min/d of sedentary time [17], and objective estimates in similarly aged children living elsewhere include 540 min/d in a multi-centre European study [7] and 480 min/d in America [23]. Besides important methodological differences (monitor type, placement, season of monitoring, data modelling and reduction), these discrepancies are likely also explained by variation in sample characteristics (including geographic location, urban–rural mix, maturation, fitness levels). The same factors may also explain why some studies have conveyed considerably different MVPA estimates to those reported herein (mean 44.8 (95% CI 39.4 to 50.9) min/d), and why in this study light intensity physical activity was more prevalent than time spent sedentary. Regardless of the absolute differences, it seems that sedentary time is high in contemporary adolescents, and higher in girls compared to boys [7,8,17,22,23].

### Patterns of physical activity and sedentary time

The wider literature has commonly shown that weekends are associated with lower objectively-measured physical activity (typically MVPA) in adolescents of both gender [8,10,11,18,49], thereby agreeing with our general observation in boys and girls of lower activity at weekends. We further found that in both genders Sunday was the least active day of the week. The evidence-base for objectively-measured sedentary time is less consistent than that for physical activity. Some studies have reported similar levels of sedentary time on week and weekend days [22,24], whilst others have found that boys and girls are less sedentary at the weekend [8,18]. We found that although boys were more sedentary during waking hours on both weekend days, girls were actually less sedentary on Saturday and more sedentary on Sunday compared to during the week. In partial agreement, the Health Survey for England found that girls’ sedentary time was approximately 15 min/d lower on weekends relative to during the week, but they found no apparent differences in boys [17]. Clearly, additional studies are needed to investigate weekday-weekend differences in sedentary time, and these studies should include gender as a potential effect modifier.

A recent review summarising the evidence for seasonal variation in childhood activity (as measured by accelerometry) concluded that UK physical activity levels are consistently higher in summer [50]. The current data corroborate these findings, and specifically indicate that seasonal patterns exist throughout the school year for light and moderate intensity physical activity. In contrast to the abundance of data for physical activity, seasonal variation in adolescent sedentary behaviour has rarely been researched. Nilsson et al. [8] found that sedentary behaviour in 15-year-olds from the European Youth Heart Study (EYHS) was not influenced by season of measurement, but their study included only spring, autumn, and winter. This study may be the first to show that during school terms sedentary time is higher in spring compared to summer in boys and girls of adolescent age. It is unfortunate, nonetheless, that we possessed few observations from the colder months (only 250 measurement days (10.5%) were in December-February) and that no data were collected in August which is one of the warmest months of the year in the UK. Future studies should work outside the confines of the school timetable and attempt to observe adolescents in all months of the year.

There remains considerable uncertainty regarding the association between SES and physical activity in youth (largely owing to wide variation in measurement of both parameters) but most studies incorporating area-based constructs of SES have failed to find a relationship with self-reported physical activity [51]. With objective data, however, we found that children living in middle SES areas engaged in more light intensity physical activity and less sedentary time than children in low SES areas. Hypothetically, this could be attributed to higher SES areas being more activity-friendly and thus encouraging light activity, but we found no evidence for behavioural differences between low and high SES areas. It is possible that by drawing inferences about individual behaviours from a group level SES variable we may have biased associations to the null by introducing misclassification errors [51]. That said, two recent investigations with objective measures of MVPA and sedentary time similarly reported no association between either variable with SES measured at the individual level (household income and maternal education) [7,9]. Additional research investigating compound SES constructs and their relation to objectively-measured physical activity is warranted.

In agreement with a large number of cross-sectional studies that have measured physical activity objectively and mainly used proxy measures for body fatness (BMI or BMI *z*-score) [52], the current study found that overfat adolescents were less vigorously physically active compared to adolescents with body fat levels <85^th^ percentile. The inverse association between body fatness and vigorous intensity physical activity is a common observation [53-56].

### Prevalence and factors associated with physical inactivity (<60 min MVPA/d)

Current physical activity guidelines in the UK specify that for maintenance of general health adolescents should participate in at least 60 min MVPA/d [4]. It is further advised that extended periods of sedentary time should be minimised but due to insufficient evidence a precise limit for daily sedentary time is not given. We therefore performed a logistic regression to identify risk factors associated with physical inactivity (<60 min MVPA/d).

Inactivity levels were found to be modest for boys and higher for girls; on average over the course of observation close to one-third of boys were by definition inactive compared to over two-thirds of girls. In boys, the odds of physical inactivity were higher at the weekend; compared to weekdays boys were 77% more likely to be inactive on Saturday and 2.8 times more likely to be inactive on Sunday. Girls were also 3 times more likely to be inactive on Sunday compared to weekdays. This suggests certain periods may be better targets for increasing physical activity levels, e.g., weekends in boys. However, our results strongly indicate that girls may have the most to gain from a physical activity intervention, as across all parts of the week they were more likely to be inactive than boys. Interventions which promote physical activity exclusively in girls have been trialled and those with robust methodology often show favourable, albeit modest, outcomes [57].

The main conclusions of our logistic model were not changed if the definition of physical inactivity was altered to explicitly include a vigorous component (<60 min MVPA/d and/or <15 min vigorous intensity physical activity per day [56]), as has been endorsed by activity guidelines [4]. However, the alternative definition caused the prevalence of inactivity to increase (boys: 54.0% inactive; girls: 85.4% inactive), and upon introduction of the vigorous component there was some evidence that overfat participants were more likely to be physically inactive compared to normal fat participants (OR = 1.68, 95% CI 0.99-2.86, *p* = 0.054). When vigorous activity was included there were also no inactivity differences between types of days in girls (*p* ≥ 0.095). This could indicate that school-based interventions may be the most efficient method of increasing girls’ health-related physical activity, as they can be targeted en masse in settings that possess readily-available facilities that can be used to encourage activity.

### Strengths and weaknesses

This study has several strengths including a relatively large sample size and an objective measurement of physical activity, which was used to derive not only an estimate of PAEE but also physical activity intensity and sedentary time. Our objective monitoring technique, which combined biomechanical and physiological signals, has been shown to yield physical activity intensity estimates with higher accuracy compared to accelerometry alone [26,27] and was worn continuously for 24 hours per day thus limiting missing data and associated biases. One of the difficulties experienced with continuous monitoring protocols is that sedentary time and sleep can be hard to distinguish from one another, potentially leading to confounded associations for sedentariness [24]. However, this study incorporated self-reported sleep information to aid separation of such behaviours, which is likely to have increased validity [32]. All individuals with one or more valid days of activity were included in the current study to maximise sample size and minimise selection factors that could have introduced bias. On average, each participant contributed 2.9 days of data; this is equivalent to the minimum recommended observation period for reliable assessment of habitual physical activity in youth [58]. A possible limitation is that mid-week days were under-represented (valid data were available for 56 Wednesdays and 98 Thursdays only), but unlike other European countries UK school children do not have free mid-week afternoons and the UK curriculum does not specify set days for physical education classes. For these reasons, it is believed that our weekday data (mainly derived from Monday, Tuesday and Friday observations) offer an adequate representation of activity performed over the entire UK school week.

Body composition data indicate that the final study sample (*n* = 825) had lower weight (Boys: 57.8 kg vs. 63.9 kg; Girls: 53.9 kg vs. 59.6 kg) and BMI (20.1 kg/m^2^ vs. 22.0 kg/m^2^) compared to the average 15-year-old child living in England at the same time that measurements were taken [59]. The final sample also exhibited a lower proportion of overweight and obese adolescents compared to the regional average (22.6% vs. 25.5%) when based upon BMI reference data [60,61]. In addition, compared to the original ROOTS’ participants who were not eligible for this study due to missing data (*n* = 378), the 825 adolescents who opted into the wave 1 physical activity testing regime and provided valid free-living data had lower wave 0 fat mass index (median 4.2 (IQR 3.1) kg/m^2^ vs. 4.5 (3.3) kg/m^2^, *p* = 0.02). Each of these features indicate some degree of selection bias related to body fatness, and suggest that the levels of physical activity reported herein may be overestimated compared to the true level in the source population. Reassuringly, however, adjusting our activity means to the level of the average BMI of 15-year-old children living in England in 2006 (22.0 kg/m^2^) caused a reduction in MVPA estimates of only 4.4 min/d (<11% of the mean).

The homogenous sample minimised the potential for confounding effects of the observed associations, but on the other it limited our capacity to discern whether or not (in) activity patterns differed by age, pubertal status, and ethnicity. It may also mean that the results are only representative of White and pubertal adolescents who are residing in peri-urban parts of the UK. Nonetheless, most of our findings for physical activity volume and time spent in MVPA concur with the established literature. A final weakness refers to the cross-sectional study design, which renders our interpretation of the association between fatness and activity challenging. Prospective studies are necessary to elucidate the direction of association between these variables. Repeated data on the same participants over the course of a school year would have also strengthened our seasonal description of activity levels.

## Conclusions

Boys are more physically active than girls at all intensities above a sedentary level and both genders predominantly engage in light intensity physical activity. The emerging concept of investigating the health effects of light intensity physical activity is therefore warranted. PAEE, physical activity intensity and sedentary time differ according to time-related factors including type of day (week or weekend) and school term, and according to area-level SES and body fatness, but the overwhelming factor that is associated with physical inactivity (<60 min MVPA/d) is female gender. If UK guidelines are sufficient for maintaining normal growth, development and health in childhood, adolescent girls should be the primary recipients of interventions that are designed to increase physical activity levels.

## Consent

Written informed consent was obtained from the participant and the participant’s guardian/parent/next of kin for the publication of this report.

## Abbreviations

MVPA: Moderate-to-vigorous intensity physical activity; PAEE: Physical activity energy expenditure; SES: Socio-economic status.

## Competing interests

The authors declare that they have no competing interests.

## Authors’ contributions

IG and UE designed the research. VD, KC and CLR helped to coordinate and conduct the research. KWe assisted PJC in processing of activity data. PJC conceived the study, performed all additional statistical analyses, and drafted the manuscript. SB designed the research, conceived the study and helped draft the manuscript. All authors read, revised and approved the final manuscript.
